# The functions of leg muscles, structures and mechanisms in running

**DOI:** 10.1098/rsbl.2024.0260

**Published:** 2024-08-07

**Authors:** James R. Usherwood

**Affiliations:** ^1^ Structure and Motion Laboratory, The Royal Veterinary College, Hatfield, North Mymms AL9 7TA, UK

**Keywords:** run, linkage, gait, muscle, Lombard’s paradox

## Abstract

The actions of the major human leg muscles are well established; however, the functions of these muscle actions during steady running remain unclear. Here, leg structures and mechanisms are considered in terms of their functions in meeting the task of a vehicle acting as an effective machine, supporting body weight during translation with low mechanical work demand and in supplying mechanical work economically. Legs are modelled as a sequence of linkages that predict muscle actions and reveal the varying muscle functions within the integrated leg. Work avoidance is achieved with isometric muscles and linkages that promote a sliding of the hip over the ground contact, resulting in an approximately horizontal path of the centre of mass. Economical work supply requires, for muscle with constrained power, shortening over the entire stance duration; this function is achieved by the hamstrings without disrupting the linkages resulting in work avoidance. In late stance, the two functions occur through coactivation of antagonistic muscles, providing one answer to Lombard’s paradox. Quadriceps and hamstring tensions result in opposing moments about both hip and knee joints, but by doing so perform the independent yet complementary roles of work avoidance during translating weight support and economical work supply.

## 1. Introduction

The actions of the major human leg muscles have been thoroughly described. In the static case, actions as flexors or extensors are simply determined by their lines of action past each joint, in some cases confirmed by electrical stimulation (e.g. [1]), and their roles described as agonist or antagonist. During gait, muscle actions can be determined through external force measurements and inverse dynamics. While it is rarely possible to measure the contributions of multiple muscles acting about the same joint directly, these can be estimated assuming suitable muscle properties and computer minimization of some gross cost function [2,3]. Muscles can be described as motors, brakes and struts, with actions often changing over the gait cycle and varying with different speeds, inclines and loads.

In order for the function of the muscle actions to be understood, it has long been appreciated (e.g. [1]) that these actions have to be considered in concert with the actions of other muscles, and as they relate to the integrated performance of the whole organism. One approach to this uses the concept of induced accelerations [2,3]. The method applies a computer musculoskeletal model to consider the outcome if a given muscle was—for an instant—absent. The predicted centre of mass accelerations can be resolved vertically and horizontally to infer muscle contributions to weight support and propulsion. However, does this method of momentary virtual oblation actually reveal the ‘function’ of a muscle? A structural column or a horse hindlimb with stay mechanism engaged highlight that weight *could* be supported entirely without muscle tension. Also, a toppling chimney or inverted pendulum demonstrates that propulsion accelerations *could* be achieved entirely without muscle action. Interpreting muscle function by modelling what would happen if it momentarily was not there, while interesting, does not necessarily indicate why the muscle was there, doing what it was doing—its *function*—in the first place.

Here, the leg is treated as a machine—a vehicle with a minimum task of supporting a load as it translates. One classic definition of a machine ([4]; refer to [5, p.28]) is ‘a collection of mechanisms arranged to transmit forces and do work’. Broadly, an effective machine is one that performs a task with low work demand and which enables whatever work that is demanded to be supplied economically. So, the task of a human leg is assumed here to be firstly that of a vehicle: to support body weight during translation, and the two demands—work avoidance and economical work supply—are assumed to be fundamental to the integrated human leg design and function in running.

**Figure 1.**
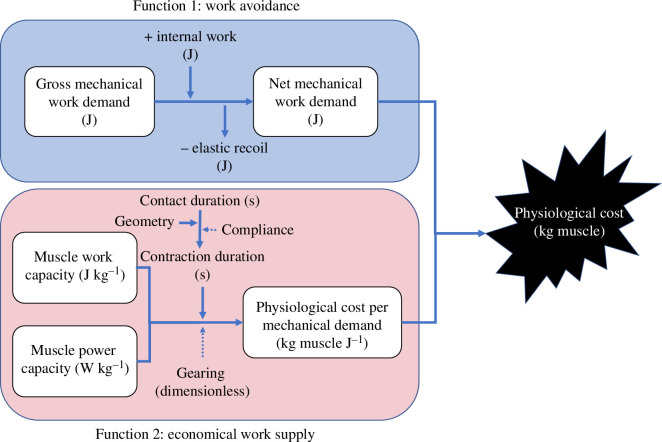
Overview of the fundamental functional demands in minimizing cost of running: minimize mechanical work demand and minimize the physiological cost of performing the work that is demanded.

The relevant physiological cost parameter is assumed to be muscle size: big muscles are very costly to grow, maintain, carry and oscillate. Importantly, even if massive muscles are maintained for reasons other than steady running ([6] highlight jumping), muscle is physiologically costly to activate: anatomy and gait that allow a smaller volume of muscle to be activated for the demands of steady running would still be beneficial. The sequences of linkages proposed here, and their associated muscle actions, are described in their functional capacity to achieve the task of weight-bearing translation while minimizing muscle mass by maximizing both work avoidance and economy of work supply (figure 1).

## 2. Fundamental functional demand 1: work avoidance

Engineered vehicles exploit mechanisms—wheels, skis, etc.—that avoid mechanical work demand by keeping weight-supporting forces (vertical) perpendicular to the motion (on level ground, horizontal). Hindlimb ground reaction force vectors in reptiles [7–9]; quadrupedal mammals [10–12] and humans (e.g. [13]; electronic supplementary material, text and video 1) pass ahead of the hip in early stance, through the hip around midstance and behind the hip in late stance. While there are measurable horizontal forces—and so fluctuations in mechanical energy of the centre of mass—these are lower than would have been the case with forces projected through the hip joint centre; somehow, to some degree, the body effectively ‘slides’ over the foot, avoiding a component of the gross mechanical work demand (figure 1).

In animals, some component of the gross mechanical work demand may also be avoided at the level of the muscle through elastic recoil, predominantly of tendons. However, this is neglected here to maintain focus on the linkage principles.

For the gross work avoidance to also result in net muscle work avoidance, the mechanism cannot rely on simultaneous positive and negative muscle powers. Muscles that are either isometric when under tension, or not under load when changing length, do not have a mechanical work demand. A range of linkage possibilities has been described for sprawled [9] and parasagittal animal legs [12] that result in a ‘sliding’ weight support, with the capacity to avoid a large proportion of the mechanical work that would otherwise be demanded from an axially loaded leg. The linkages support horizontal motion of the hip with predominantly vertical ground reaction forces thereby avoiding mechanical work demand, with structures and mechanisms recruiting various muscles that remain isometric when under load, preventing muscle mechanical work. Isometric muscle can achieve higher stresses than shortening muscles (see below), and so are less costly than shortening muscles per unit force. In addition, a muscle functioning to simply lock a joint could be highly geared (have a high muscle mechanical advantage), further reducing the amount of muscle required per unit moment (torque) compared with a powering, shortening muscle. It is assumed here that the physiological cost associated with muscle tension is negligible compared with any demands for muscle work; the details of musculoskeletal geometry are not required—if a muscle can lock a joint, or contribute to a four-bar linkage, while isometric, it is assumed to do this without cost.

A ‘linkage’ in this context is a structure or mechanism consisting of links (muscles and/or tendons, bones) connected by freely moving joints. The ‘legs as linkages’ approach differs from conventional anatomy and biomechanics in considering as joints not only the bone–bone connections but also the connections between tensile elements (muscles and/or tendons) and their sites of origin and insertion. It also puts particular initial focus on the consequences of links that are *not* changing length rather than those that do.

## 3. Fundamental functional demand 2: economical work supply

**Figure 2.**
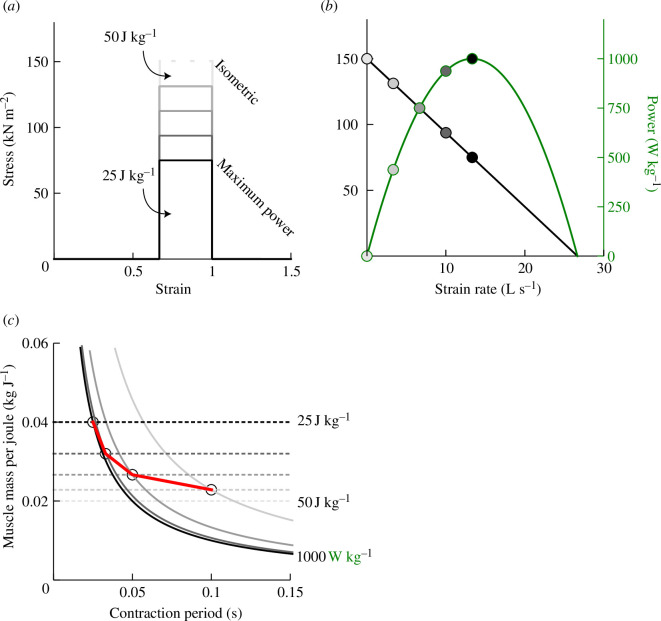
Stress–strain (*a*), stress- and power–strain rate (*b*) and their consequences in terms of muscle mass demand per joule of mechanical work (*c*) for an idealized muscle maximally capable of W* = 50 J kg^−1^ (contracting exceedingly slowly, palest grey), P* = 1000 W kg^-1^ (contracting at an intermediate speed (black)), an isometric stress of 150 kN m^−2^ and a density of 1000 kg m^−3^. The muscle parameters and properties of a rectangular stress–strain and linear stress–strain rate relationships are extremely simplifying; however, the principle appears secure: a longer contraction duration allows a smaller volume of muscle to produce a unit of mechanical work. Model lines (*c*) for the work constraint (dashed lines) are *y* = 1/W*; for the power constraint (solid curves) *y* = 1/(P*t), where W* and P* are the mass-specific work and power capacities and *t* is the contraction duration. Their intercepts (at a range of muscle stress/strain rate pairings shown with the red line) indicate the minimum muscle mass required for a joule of work for a given contraction duration.

There is clearly variation in muscle efficiency depending on loading parameters; despite the assumptions above, an isometric muscle under tension does require some metabolic input, yet performs no net mechanical work, and so is 0% efficient. Attempts to quantify the detail of muscle cost and efficiency for running require accurate musculoskeletal geometries, kinematics and muscle properties; not the approach of this paper. Instead, it is assumed that suitable ‘gearing’ prevents muscle stress, strain and strain rate from being costly in any terms other than as corollaries to their fundamental roles in providing muscle work and power. The cost function to be minimized here is simply muscle size, and the muscle size depends on the work and power demand. The size of muscle demanded for unit work supply depends critically on the duration over which the muscle contracts (figure 2; code in the electronic supplementary material). Brief contractions, given a constraint to muscle power, require a large mass of muscle to produce a joule of work; contractions over longer periods are more economical. Even with optimal gearing, briefer contractions require bigger muscles: either stresses are reduced because of higher strain rates, or strains are reduced owing to insufficient strain rates and reduced contraction duration, or both. This principle has wide-reaching implications, providing a general explanation for the scaling of posture, of top running speed, gait mechanics in children and flapping bird flight strategies (refer to [14] for a summary and [15]). While spring or tendon loading and recoil (‘compliance’, figure 1) can help matters somewhat by decoupling muscle contraction duration from the timing of mechanical power supply, both in single-shot actions (consider bows and catapults) and in cyclic gaits (e.g. Achilles [16]), they are not included in the models developed here. Instead, the focus is on gross limb design, joint and muscle action that allow muscles to contribute mechanical power for a large proportion of stance, critically without disrupting the work-avoiding geometries that minimize work demand. The analogy with a bicycle is that appropriate selection of pedal crank length and gearing can allow economical muscle work supply without altering the roundness of the work-avoiding wheel.

## 4. Linkage model development for ‘sliding’ weight support in human running

The modelling approach is to design sequential kinematic linkages with isometric links (when under load) approximating the ‘sliding’, work-avoiding, kinetic ideal. The geometries are deliberately simplified with the aim of demonstrating the principle and generality of the proposed structures and mechanisms. The purpose of these models is to reveal the functions of the various muscle actions, not to accurately report or predict kinetics or forces.

**Figure 3.**
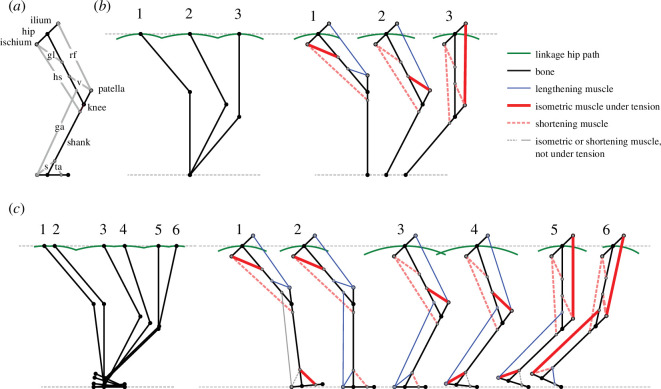
Kinematic scenarios that allow an idealized leg (*a*; for muscle names, see table 1) to support a ‘sliding’, near-horizontal motion throughout stance with three arcs given pelvis–thigh–shank (*b*) or six arcs with heelstrike and pelvis–thigh–shank–foot (*c*) leg models. A series of muscles come under isometric load (bold red), engaging a sequence of linkages that provide the structures and mechanisms resulting in horizontal motion during vertical weight support for each snapshot ((*a*) 1–3; (*b*) 1–6). Muscles that are shortening with these kinematics (pink dashed lines) have the capacity to contribute mechanical power by contracting under tension *without* disrupting the underlying work-avoiding linkages. Muscles that supply positive work throughout stance (exemplified by the hamstrings) do not contribute to the work-avoiding mechanisms but can contribute power economically: the muscle mass demanded per joule of mechanical work supplied is minimized owing to maximized contraction period.

Two models are developed (figure 3; table 1; electronic supplementary material, videos S2–S5 and code): a three-part pelvis–thigh–shank model (geometry derivations in the electronic supplementary material) and a four-part pelvis–thigh–shank–foot model of heelstrike running. The linkages support straight-line, horizontal motion through a series of snapshots. A sufficiently large pitch moment of inertia is assumed for pitching accelerations to be neglected. A constant velocity at a constant height would require zero mechanical power; this is clearly not exactly achieved by the linkage models (visible arcs remain) or by sprinting humans; however, it is assumed this state approximates both model and reality sufficiently to allow insight. Consistent with the assumption and models, empirical forces do not project from a point foot directly through the hip; instead, they project ahead of the hip in early stance and behind in late (electronic supplementary material, video S1), and the centre of pressure moves from heel to toe. Further, fluctuations in height during stance are remarkably low −2 to 3 cm for Usain Bolt (from [17]). This approximately horizontal path is achieved in the models either by sequential arcs as different joints become locked or are released or by the formation of four-bar linkages. Where possible, the linkages are kept geometrically simple (a parallelogram), allowing an intuitive appreciation of the principles of action. However, in the four-part leg, the distal four-bar requires tuning; gastrocnemius origin and insertion positions are arbitrary, but the gastrocnemius length needs to be tuned if it is to produce a suitable six-bar linkage (figure 3*c*, snapshot 6).

**Table 1.** Major leg muscle groupings and their putative roles in engaging isometrically (bold **Iso**) and forming a sequence of structures and mechanisms that facilitate economical, ‘sliding’ weight support. Lengthening muscles (eccentric: Ecc) do so under zero tension (perform no negative work) until their longest length, at which point they can engage geometrically, suggesting the possibility that reflex activation might contribute to the precise timing and sequencing of muscle tensions over the stance. Shortening muscles (concentric: Con) either do so without tension and produce no work or can contract and supply mechanical power without disrupting the work-avoiding linkage geometries. Muscles that are isometric but need not be under tension, (unbolded Iso) play no role in determining the structures or mechanisms at that instant.

(a) without foot		1	2	3				
	kinematic consequence	arc knee about foot	arc hip about foot	arc hip about knee				
gluteus (gl)	hip lock	**Iso**	Con	Con				
vastus (v)	knee lock	Ecc	**Iso**	Con				
rectus femoris (rf)	four-bar	Ecc	Ecc	**Iso**				
hamstrings (hs)	none	Con	Con	Con				

(b) with heelstrike		1	2	3	4	5	6	6+
	kinematic consequence	arc knee about heel	arc knee about ankle	arc hip about ankle	arc hip about toe	arc hip about knee	Watt’s six bar (two 4-bars)	further leg extension
gluteus (gl)	hip lock	**Iso**	**Iso**	Con	Con	Con	Con	[Con]
vastus (v)	knee lock	Ecc	Ecc	**Iso**	**Iso**	Con	Con	[Con]
rectus femoris (rf)	proximal four- bar	Ecc	Ecc	Ecc	Ecc	**Iso**	**Iso**	[?]
tibialis anterior (ta)	ankle lock	**Iso**	Con	Con	(Iso)	Ecc	Ecc	[Ecc]
soleus (s)	ankle lock	(Iso)	Ecc	Ecc	**Iso**	**Iso**	Con	[Con]
gastrocnemius (g)	distal four-bar	(Con)	Ecc	Ecc	Ecc	Ecc	**Iso**	[Con/recoil]
hamstrings (hs)	none	Con	Con	Con	Con	Con	Con	[Con]

The linkages developed here neglect the possibility of a tension element originating behind the hip, crossing the femur and inserting at the patella. This link is readily apparent in most mammals (cranial biceps femoris) and birds (iliotibialis lateralis pars postacetabularis) and would result in a ‘sliding’ retraction and passive leg shortening over early stance—an economically appealing possibility considered by Alexander [18] but rejected for humans owing to lack of anatomical evidence.

The gross kinematics of both models are, given the horizontal path, segment lengths and assumption of forward-pointing knee, inevitably broadly reasonable: the shank is near-vertical in early stance, the knee maximally flexed in midstance and the thigh goes through vertical and rotates about the knee in late stance. These kinematics are achievable with linkages that first lock the hip (gluteus isometric; figure 3*a,b*, snapshot 1), then the knee (vastus isometric; figure 3*a,b*, snapshot 2), then result in knee extension owing to the parallelogram of patella, thigh, ilium and isometric rectus femoris (figure 3*a,b*, snapshot 3). When the rectus femoris engages, forward motion of the knee is prevented as the knee-patella link is locked parallel to the ilium, and the thigh and rectus femoris arc, in parallel, about the knee and patella insertion respectively. Joint power profiles from inverse dynamics derived from external ground reaction forces are consistent with these linkages (see the electronic supplementary material): in early stance, knee power is low as the force vector passes close to the knee joint, while hip power is low as the thigh retracts slowly; later, knee power is low as the knee flexion angle is constant, and hip power low as the force vector passes close to the hip; in later stance, knee power is high positive and hip high negative, broadly cancelling, consistent with the four-bar linkage mechanism.

While the four-part leg is less constrained, the heelstrike assumption and the principle of dividing the horizontal path into many instances of weight support limit the options available; the kinematic sequence is again largely inevitable. Measured electromyography (EMG) ([19]; though refer to [20] concerning quadriceps measurements) and simulated activation [3] profiles broadly agree with those predicted here assuming that the order of intensities follows (table 1): isometric while under tension > concentric > eccentric.

## 5. Insights from the linkage-leg models

The models demonstrate the muscle actions involved in generating a series of linkages enabling the leg to perform the task of a vehicle—weight support during translation—with low muscle mechanical work demand. This provides a functional explanation for the action of muscles as they come under tension isometrically and act as tension struts: the resulting structures and mechanisms support horizontal motions and promote work avoidance.

The geometries of the work-avoiding linkages show that the distance between hamstring origin and insertion can reduce throughout stance as first the shank rotates under the knee, then the thigh and shank rotate under the hip, and finally, the thigh rotates under the hip. This means that, were the hamstring to shorten while under tension and so contribute mechanical work, it would be able to do so through the entire stance—and so, economically*—without disrupting the work-avoiding geometries*.

The two distinct fundamental demands of the muscles and their associated structures and mechanisms provide one account for ‘Lombard’s paradox’ ([21]; refer to [22] for overview): what is the function of simultaneous muscle tension in both rectus femoris and hamstrings? The two muscles are antagonists to each other, with the hamstrings imposing extension moments about the hip and flexion about the knee, and the rectus femoris the opposite. Simultaneous tension in hamstrings and rectus femoris can power an action such as standing from a seated position, but their opposing moments would appear energetically detrimental (the same motions could have been powered with single-joint muscles without antagonist muscle forces) and some benefit such as ‘joint stability’ is often asserted as functional (refer to [23]). But what is the function of co-contraction of antagonists in running? It is possible that this is a consequence of running with mechanical linkages that are adaptive for other actions—such as leaping—or incomplete separation of neural control. However, the linkage-leg models indicate the two muscles contract with two discrete functions: in late stance, the isometric rectus femoris forms a four-bar linkage resulting in passive leg extension and sliding work avoidance; the shortening hamstrings can produce work over the entirety of stance and so supply work economically. Co-contraction of the two antagonistic muscles allow the two functions to be performed *without interfering with each other*.

The geometric sequencing of linkage engagement within a stance, and the division of function between work-avoiding, trajectory-determining and economical work-supplying muscles, predicts an interplay between top-down and feedback control. If the leg can indeed be considered a ‘kinematic machine’ formed of a sequence of linkages, the hip path could be decided (top-down) on a step-by-step timeframe by controlling the trajectory-determining muscle limiting-lengths, allowing the rapid changes in muscle tension over the course of the stance to be driven by geometry and reflex (feedback) activation (refer to [24]). The division of function means that the input of work from the tension in the hamstrings need not be exquisitely controlled in terms of either amplitude or timing. Returning to the bicycle analogy: the cyclist can slowly decide how hard to pedal (analogous to hamstrings tension), and the spokes rapidly vary their tensions (analogous to gluteus–vastus–rectus femoris tensioning in the three-part model) resulting in a work-avoiding trajectory while being insensitive to the timing of the powering function, and without requiring further top-down input.

## 6. Conclusion

Leg design, joint and muscle action in human running can be viewed as a sequence of geometrically engaged and disengaged linkages, achieving the task of a vehicle—weight support during translation—alongside the demands of an effective machine—mechanical work avoidance and economical work supply. While implications of a compromise between the two demands have been explored quite broadly, accounting for the scaling of posture, bipedal and flapping gaits [14], or have been used to disregard certain potential work avoidance strategies (in human walking; [25]), the linkages described here demonstrate how the two functions can be achieved simultaneously within the same leg, but with different muscle actions, without compromising each other.

## Data Availability

The code for generating the figures coordinates are available from the Dryad Digital Repository [26]. Supplementary material is available online [27].
